# Supplementary figures and images for: Anticancer Effect of Benzimidazole Derivatives, Especially Mebendazole, on Triple-Negative Breast Cancer (TNBC) and Radiotherapy-Resistant TNBC In Vivo and In Vitro
Source: Molecules. 2021 Aug 24;26(17):5118. doi: 10.3390/molecules26175118 (PMC8433818; doi:10.3390/molecules26175118)

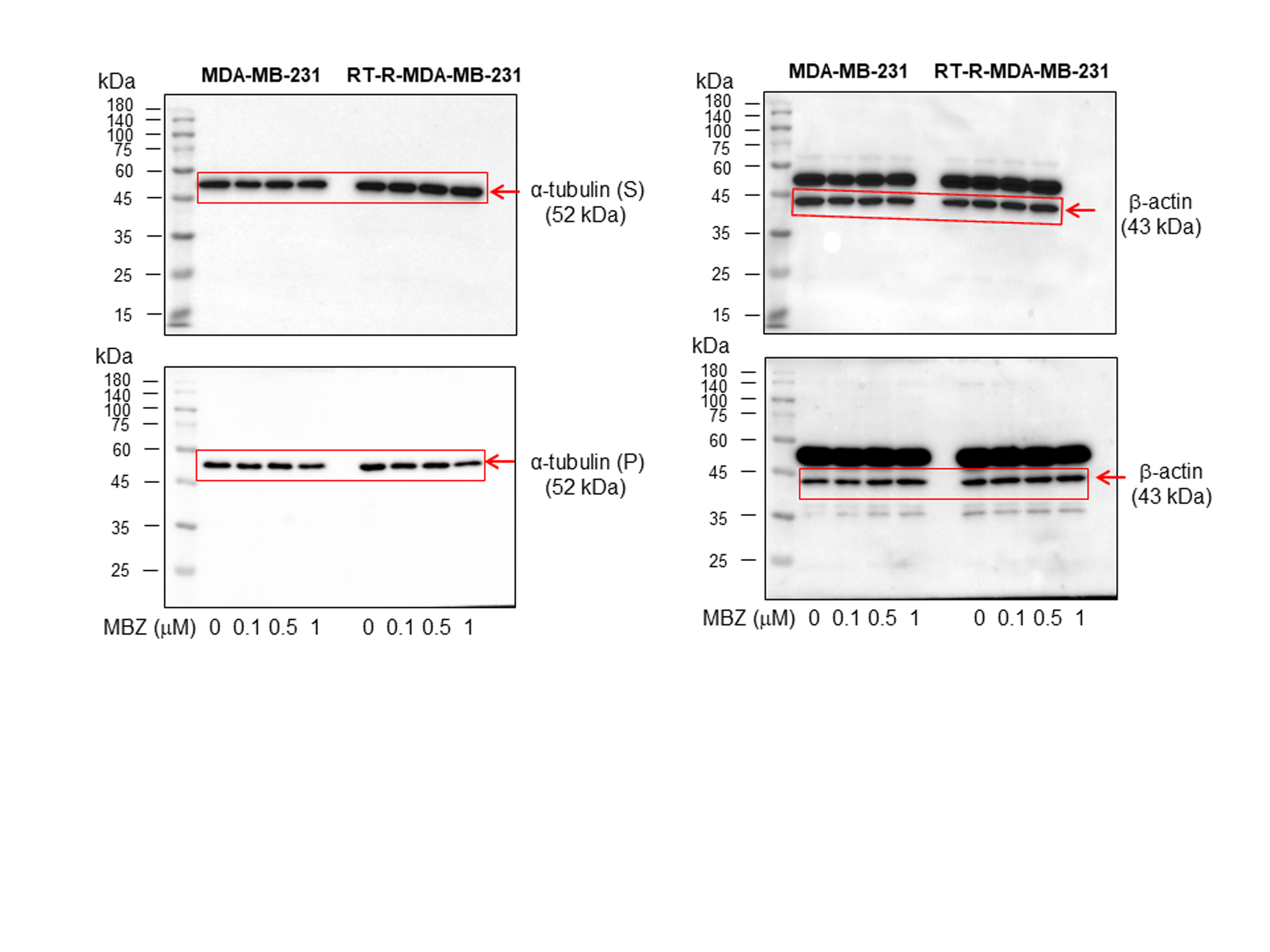

Supplement: Supplementary file 1 [file molecules-26-05118-s001.zip › Supplemental materials_Figure S1_Fig. 5b.tif]

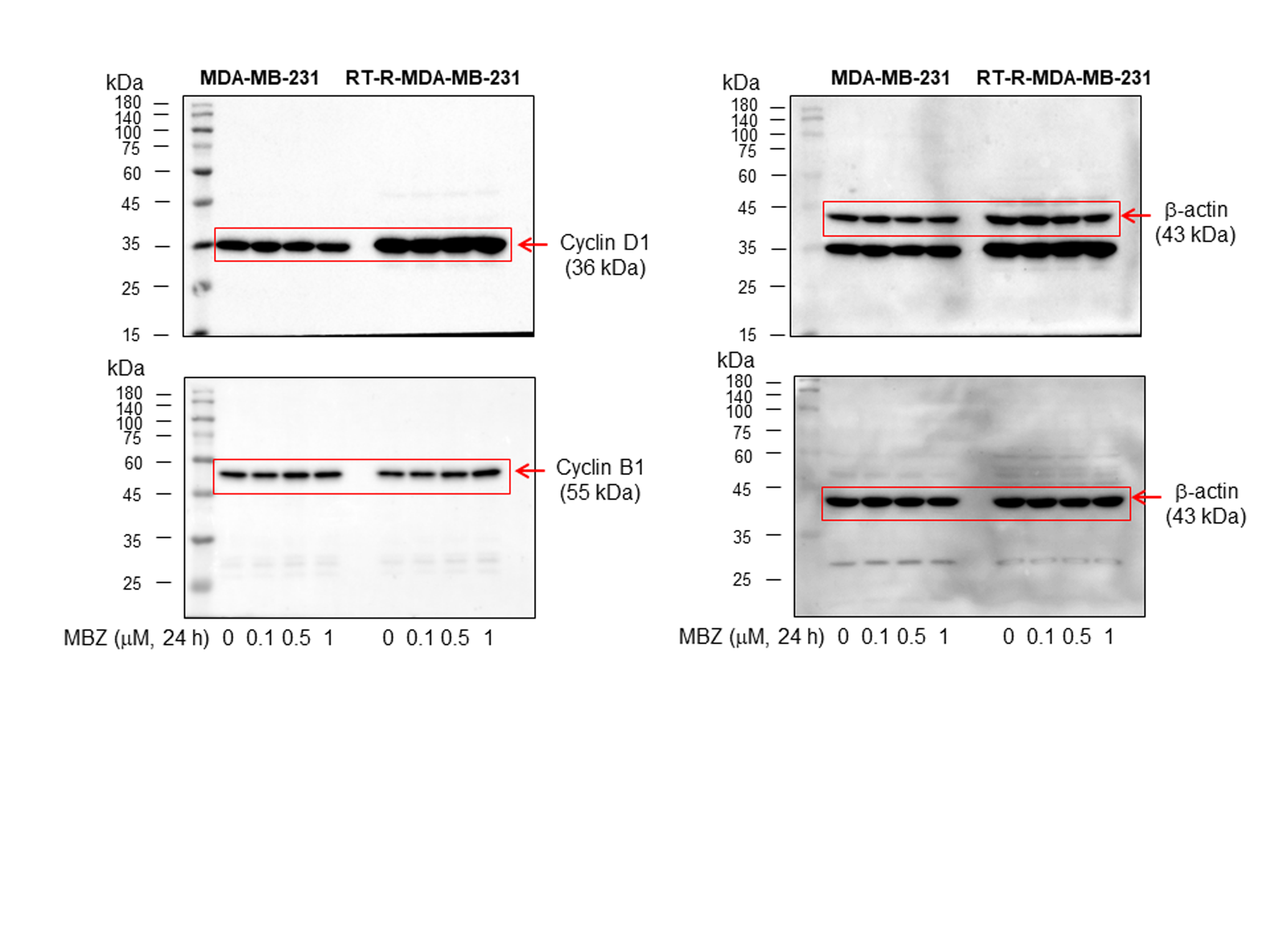

Supplement: Supplementary file 1 [file molecules-26-05118-s001.zip › Supplemental materials_Figure S2_Fig. 5c.tif]

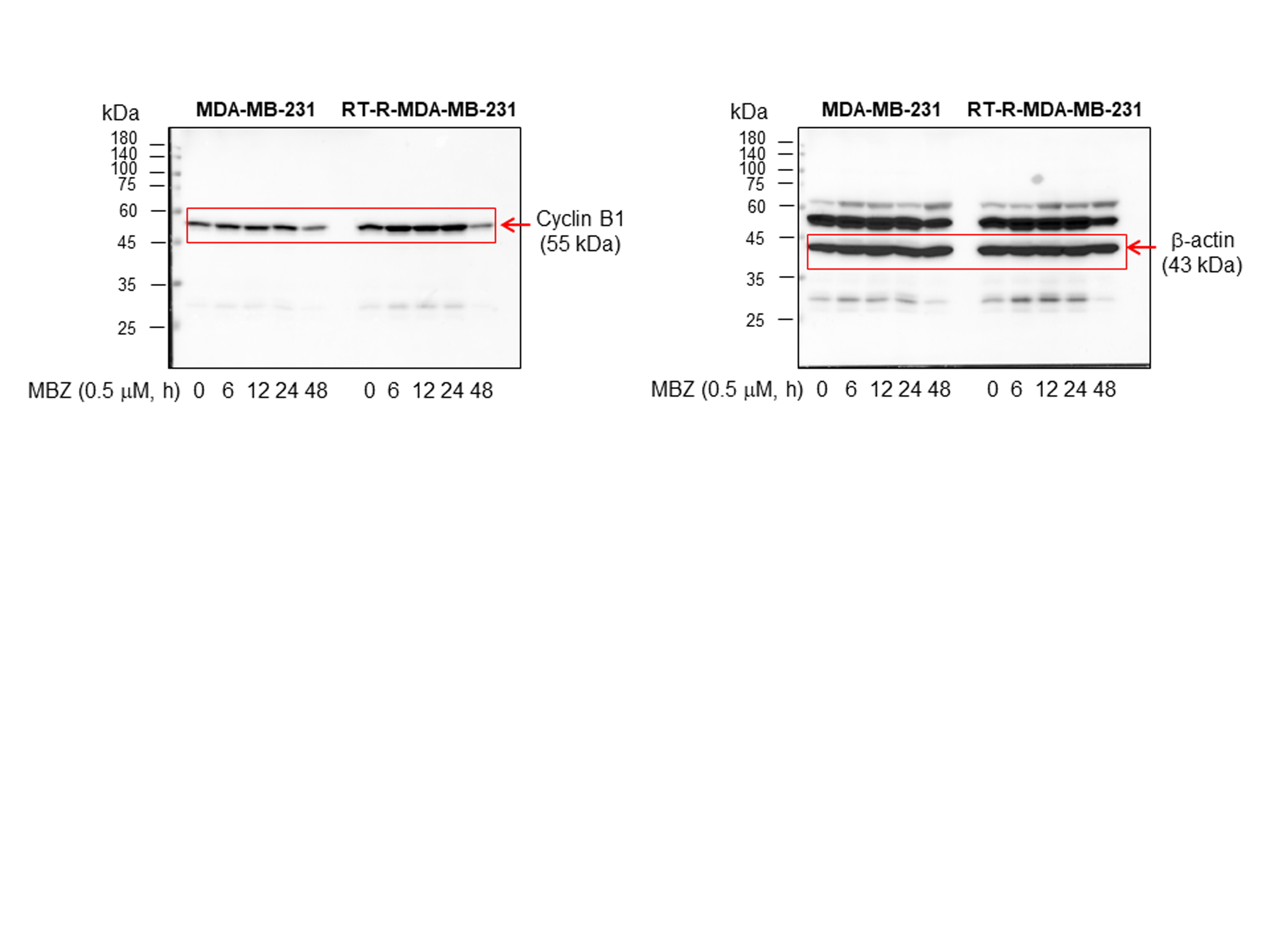

Supplement: Supplementary file 1 [file molecules-26-05118-s001.zip › Supplemental materials_Figure S3_Fig. 5d.tif]

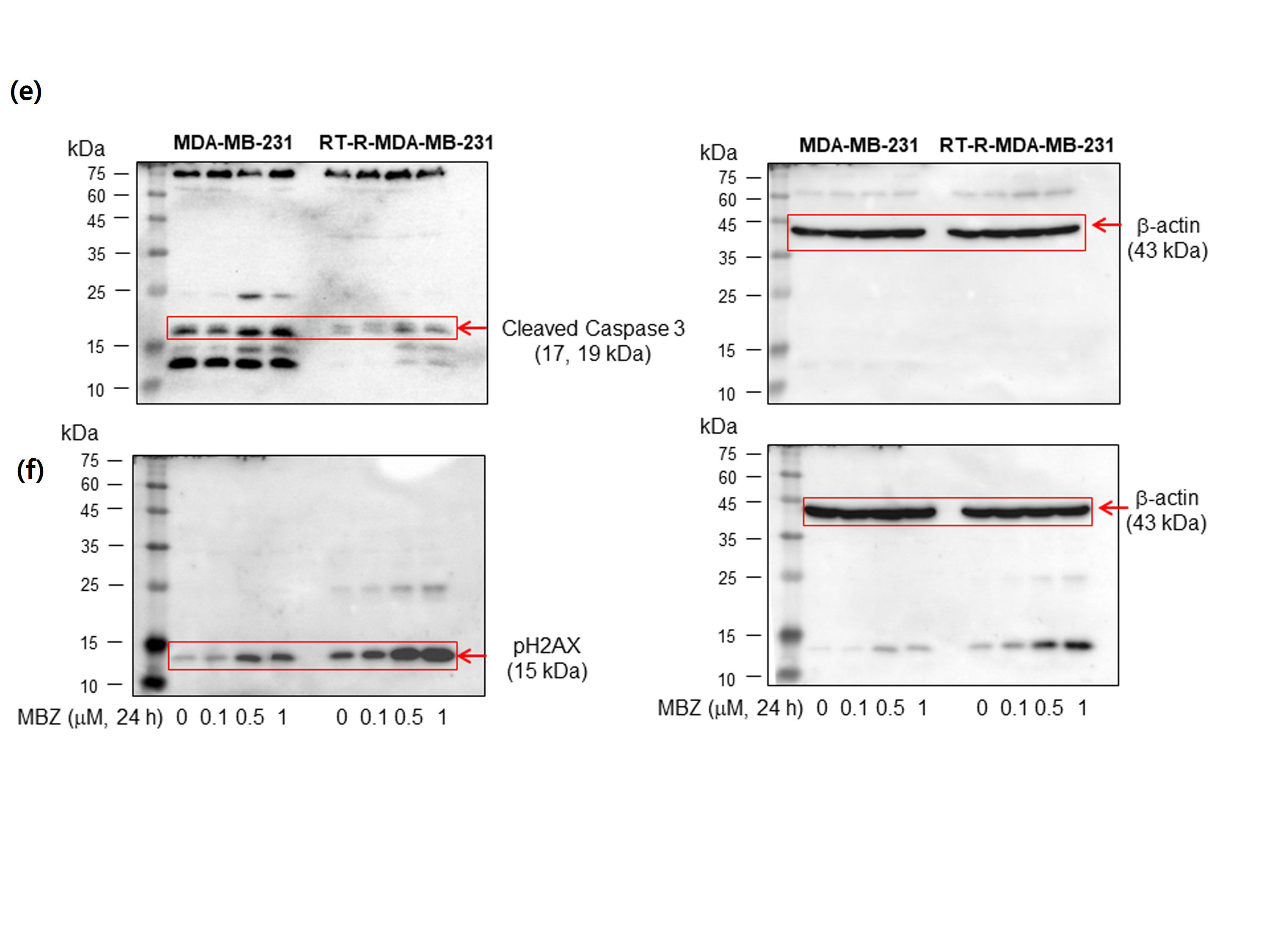

Supplement: Supplementary file 1 [file molecules-26-05118-s001.zip › Supplemental materials_Figure S4_Fig. 5ef.tif]

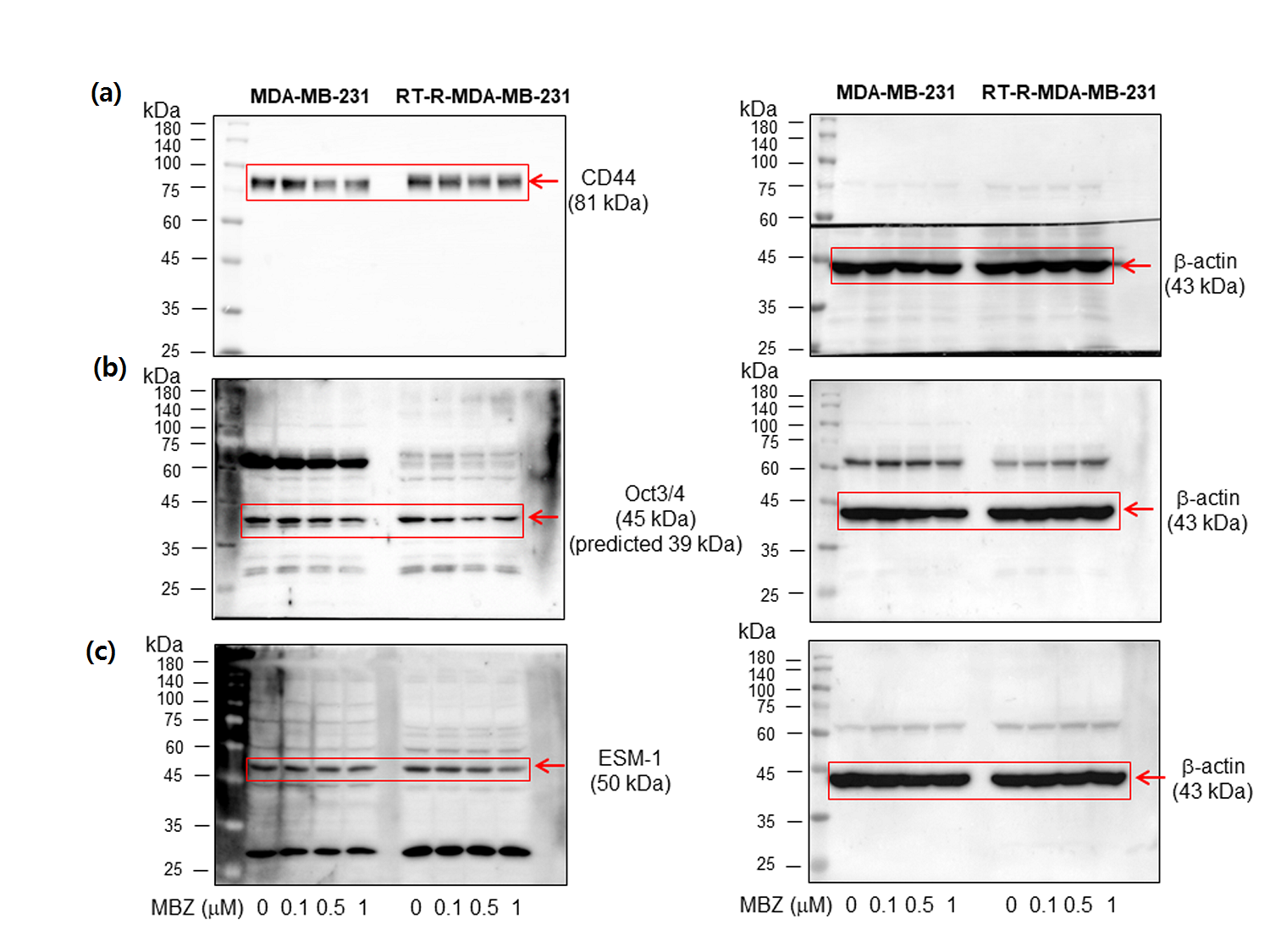

Supplement: Supplementary file 1 [file molecules-26-05118-s001.zip › Supplemental materials_Figure S5_Fig. 6.tif]
